# Putative Genes of *Pathogenesis-Related Proteins* and *Coronatine-Insensitive Protein 1* in *Ribes* spp.

**DOI:** 10.3390/plants11030355

**Published:** 2022-01-28

**Authors:** Ana Dovilė Juškytė, Ingrida Mažeikienė, Vidmantas Stanys

**Affiliations:** Lithuanian Research Centre for Agriculture and Forestry, Institute of Horticulture, Kaunas str. 30, Babtai, 54333 Kaunas, Lithuania; ingrida.mazeikiene@lammc.lt (I.M.); vidmantas.stanys@lammc.lt (V.S.)

**Keywords:** blackcurrant, defense response, gene homolog, *pathogenesis-related* genes, *coronatine-insensitive 1*

## Abstract

In response to pathogen attacks, plants activate a complex of defense mechanisms including an accumulation of the endogenous signaling compounds salicylic acid and jasmonic acid. The activity of *pathogenesis-related* genes (*PR*s) and *coronatine-insensitive 1* (*COI1*) in defense-response pathways are established in plants. The aim of this study was to identify homologs of the *PR*s and *COI1* in blackcurrants. Primers with degenerate nucleotides were designed based on the most conservative parts of *PR1* and *COI1* genes from other plants and applied for amplification of specific fragments of *PR*s and *COI1* in *Ribes* spp. Seven heterogeneous sequences of *PR* with a diversity of 66.0–98.3% at nucleic acid level were found. The phylogenetic analysis revealed the dependence of *R. nigrum* *PR* homologs on the *PR1* and *PR6* families. Four heterogeneous sequences of *R. nigrum* *COI1* with an identity of 95.9–98.8% at nucleic acid level were isolated. Specific primers for newly detected genes’ homologs were designed in this study and could be useful for evaluating the defense response to pathogen attacks in blackcurrants.

## 1. Introduction

Blackcurrant (*Ribes nigrum* L.) is an economically important berry, widely grown in the temperate climate zones of Europe, Asia, New Zealand, Australia, and North America. Fruits and other vegetative parts of *Ribes* spp. have a rich nutritional composition, so they are widely used in food, pharmacology, and the cosmetics industry [1]. Blackcurrant plantations worldwide are adversely affected by various pests, pathogens and diseases. The breeding of resistant cultivars is a top priority; however, the application of new biotechnological methods in *Ribes* breeding is limited by a lack of genetic knowledge and unsequenced genome [2]. To this day, only a few genes and molecular markers specific to pathogen and pest resistance have been established in some *Ribes* species [3,4,5,6,7]. This is why genetic mechanisms of resistance in *Ribes* genus are unknown, and the metabolic pathway involved in defense response after pest or pathogen infection is limited [8].

Plants have developed a complex defense system against biological agents of various scales, from microscopic viruses to phytophagous insects. The components of this immune system rely on their ability to recognize pathogen molecules, carry out signal transduction and respond defensively through pathways involving many genes and their products. The relevance of defense-related hormones such as salicylic acid (SA), jasmonic acid (JA) and ethylene (ET) as primary signals in the regulation of signal transduction cascades in plant defense has been widely investigated [9]. Once pathogens overcome mechanical barriers to infection, plant receptors initiate signaling pathways, stimulating the defense-response genes’ expression [10]. Inducible defenses include a rapid oxidative burst, accumulation of elevated levels of endogenous signaling compounds, induction of defense-related genes and production of antimicrobial enzymes [11].

Pathogenesis-related proteins (PRs) are plant species that specific proteins induce by biotic stress, and they play an important role in plant defense against pathogenic fungi, virus, bacteria, and insects. In addition to their protective function, they are also involved in plant hormone regulation and cell development [12]. Proteins encoded by *PR* genes have been classified into 17 families according to sequence homology, isoelectric points, reaction with specific antisera and mRNA probes [13]. Most *PR* genes are induced through the action of SA and JA signaling compounds and possess antimicrobial activities *in vitro* through hydrolytic activities on cell walls, contact toxicity and perhaps an involvement in defense signaling. *PRs* are associated with systemic acquired resistance (SAR) and can act locally on pathogens in the cell or through enzymatic activity [14,15].

Plant pathogens deploy an array of virulence factors, such as phytotoxin coronatine (COR), to suppress host defense and promote pathogenicity [16]. COR can mimic bioactive JA compounds in plants, thus targeting JA receptors to *coronatine insensitive 1* (*COI1*) gene expression and inhibiting the SA-signaling pathway of defense [17]. The JA-responsive defense-related gene *COI1*, which has been identified as insensitive to COR *A. thaliana* mutants, is required for resistance to insect herbivory and resistance to pathogens [18]. In addition to its protective function against pests, *COI1* is also involved in other plant physiological processes: growth inhibition, reproduction and regeneration [19].

The aim of this study was to identify homologs of the *PR*s and *COI1* genes in *Ribes* spp. emphasizing the *R. nigrum* genome.

## 2. Results

### 2.1. Development and Application of PR and COI1 Primer Pairs

**Figure 1:**
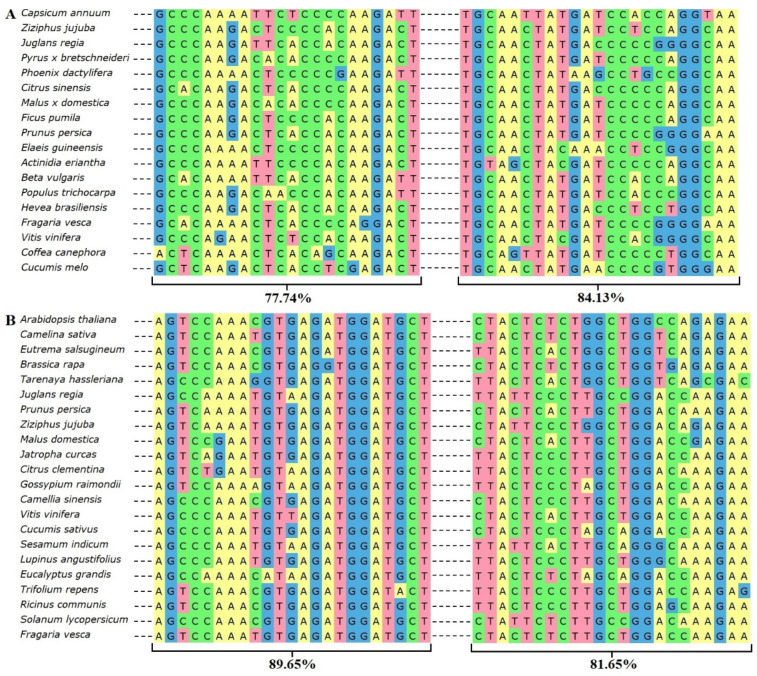
Alignments of the most conservative sites of *PR1* (**A**) and *COI1* (**B**) genes in various plants. Accession numbers of presented plants in Figure 1 are available in Table S1.

One of the goals in our study was to design oligonucleotide primers suitable for *Ribes* spp. that are distinct from other agricultures in taxonomy. The most distinctive sequences from rarer woody garden plants and other dicotyledons were chosen for alignments and primers design (Figure 1).

*PR* genes have been extensively studied in plants, and families from *PR1* to *PR17* are recognized. The genome of *Ribes* spp. has been insufficiently investigated, and this has led to difficulties in biotechnological approaches. The PRPd primer pair (Table 1) with degenerate nucleotides was designed according to data on multiple nucleic acid alignment of *PR1* genes in 18 plant species (Figure S1). The sites with the highest homology were located from 66 to 88 and from 435 to 458 nucleic acids in the *C. annuum* (the NCBI database accession number is AF053343.2) sequence of the whole *PR1* gene (Figure 1A). The sites’ similarity of sequences selected for forward and reverse primers design were 77.7% and 84.1%, respectively. The COId primer pair with degenerate nucleotides (Table 1) was designed comparing *COI1* gene sequences from 22 plant species according to the NCBI database (Figure S2). The primer pair for detection of *COI1* in *Ribes* spp. was designed from the most conservative sites of the gene, from 1383 to 1406 and from 1712 to 1735 nucleic acids., according to the *A. thaliana* sequence (NM_129552.4) (Figure 1B). The sequence homology at the primer sites was 89.7% for forward direction and 81.7% for reverse direction among 22 plant species.

**Figure 2:**
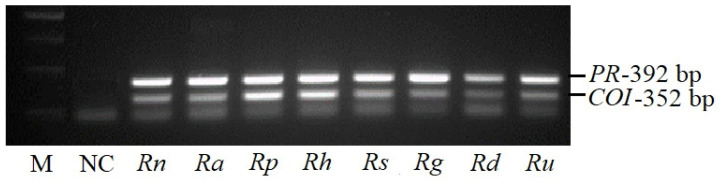
Amplified PCR fragments obtained with primer pairs PRPd and COId in *Ribes* species. M—size standard 250, 500, 750, 1000 bp; NC—negative control; *Rn*—*R. nigrum*; *Ra*—*R. americanum*; *Rp*—*R. pauciflorum*; *Rh*—*R. hudsonianum*; *Rs*—*R. sanguineum*; *Rg*—*R. glandulosum*; *Rd*—*R. dikusha*; *Ru*—*R. uva-crispa*.

These newly designed primer pairs with degenerate nucleotides (Table 1) were applied for detection of *PR* and *COI1* homologs in various *Ribes* species by PCR (Figure 2). The entire lengths of *PR1* and *COI1* in the plants were approximately 500 and 1780 nucleotides, respectively (according to the NCBI database, Table S1). The primer pair PRPd was suitable to amplify 392 bp length product from the middle of the *PR* gene by PCR. The primer pair COId flanked part of the *COI1* gene at the 3′ end, from 1383 to 1735 bp.

The PCR application of the primers PRPd and COId was performed in eight *Ribes* species: *R. nigrum* cv. Didikai, *R. americanum*, *R. pauciflorum*, *R. hudsonianum*, *R. sanguineum*, *R. glandulosum*, *R. dikusha* and *R. uva*-*crispa*. Specific size fragments for the *PR* (392 bp) and *COI1* (352 bp) were amplified in tested species (Figure 2). For further genetic study, specific products of *COI1* and *PR* genes from *R. nigrum* (Figure 2, column 3) were excised from purified agarose gel, and DNA samples of 20 plasmids after transformation were sequenced.

### 2.2. Multiple Sequence and Phylogenetic Analysis of PR Homologs of R. nigrum

**Figure 3:**
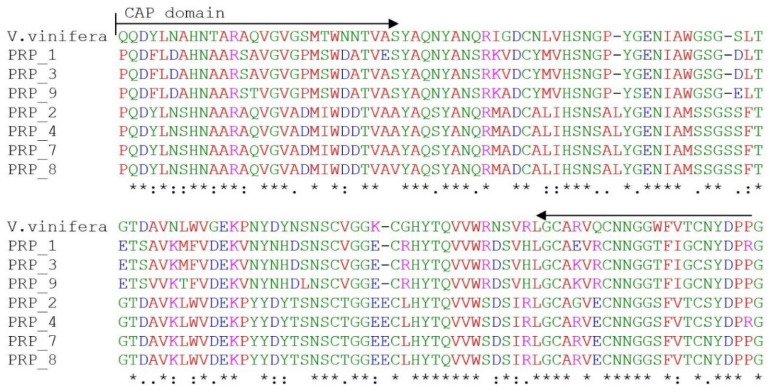
A multiple alignment of seven *R. nigrum* isolates and part of *PR1* gene of *V. vinifera* (whole *PR1* is 160 amino acids, according to the accession number XP_002273416.1). The underlined sequence marks the CAP domain. The comparative sequence is the amino acids for residues 28–152 of the protein. Identity of amino acids: *—identical, :—conservative, .—semi-conservative, space—non-conservative.

Seven heterogeneous sequences of *PR* were identified in the blackcurrant mRNA: PRP_1, PRP_2, PRP_3, PRP_4, PRP_7, PRP_8 and PRP_9 (accession numbers in the NCBI database are OK625407–OK625413) (Table S5). Partial sequences of *PR* identified in this study were in position 28–152 amino acids, compared with the whole gene of *V. vinifera* (XP_002273416.1) (Figure 3). The substitutions and deletions among the sequences are shown in Figure 3. All *PR* isolates of *R. nigrum* had mutations at the amino acid level. The percent identity among the nucleotide sequences obtained in *R. nigrum* ranged from 66.0 to 98.3%, and from 63.2 to 98.5% at amino acid level (Table S3). A multiple sequence alignment with *V. vinifera* showed that the *R. nigrum PR* isolates had 54.7% identical and 13.3% conservative amino acids (Figure 3). According to deletions of amino acids and substitutions of semi-conservative (10.9%) or non-conservative (21.1%) amino acids, two groups of *R. nigrum* isolates were visible: PRP_1, PRP_3 and PRP_9, and PRP_2, PRP_4, PRP_7 and PRP_8. The CAP superfamily domain (Cysteine-Rich Secretory Proteins, Antigen 5, and Pathogenesis-Related 1 Proteins), which is underlined in Figure 3, shows the dependence of newly detected blackcurrant isolates on the *PR* gene family.

**Figure 4:**
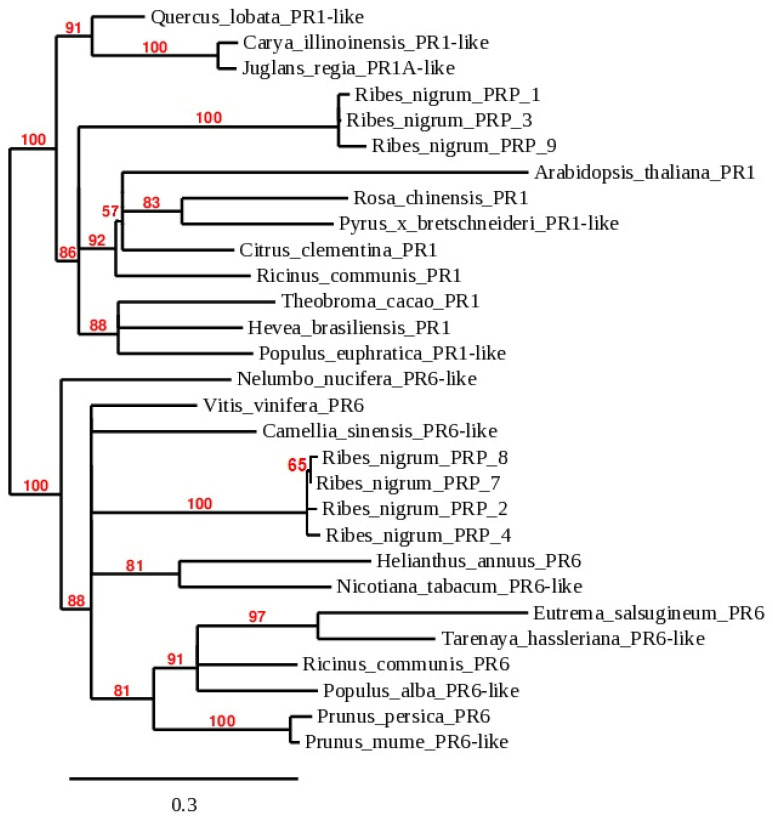
Phylogenetic dendrogram of *PR* genes constructed according to nucleotide sequences of 22 plant species. Accession numbers of newly obtained *PR* sequences of *R. nigrum* in our research are OK625407–OK625413 (Table S5); accession numbers of other plants in Figure 4 are available in Table S2.

Phylogenetic analysis of seven *PR* homologs of *R. nigrum* was conducted in a general context of 22 plants (Figure 4). The *PR* homologs were divided into two distinct branches, as *PR1* and *PR6* families at 100.0% bootstrap. A separate branch consisted of three *R. nigrum* isolates—PRP_1, PRP_3 and PRP_9—that are likely to be *PR1* homologs. Another four isolates—PRP_8, PRP_7, PRP_2 and PRP_4—were grouped in another branch of phylogenetic tree and showed a clear dependence on the family *PR6*. Isolates PRP_7 and PRP_8 of putative gene *PR6* were genetically distinguished at 65.0% bootstrap. Newly identified *PR* isolates in blackcurrants appeared as members of the *PR1* and *PR6* families, although they also showed the uniqueness of the *Ribes* genus from other plants at bootstrap 100% in both branches of the phylogenetic dendrogram (Figure 4).

### 2.3. Multiple Sequence and Phylogenetic Analysis of COI Homologs of R. nigrum

**Figure 5:**
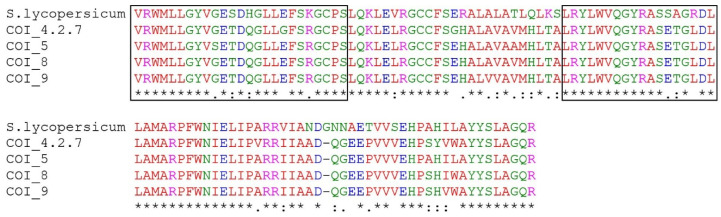
A multiple alignment of four *R. nigrum* isolates and part of *COI1* gene of *S. lycopersicum* (whole *COI1* is 603 amino acids, according to the accession number NP_001234464.1). Sequence part in boxes indicate two leucine-rich repeat (LRR) regions. The comparative sequence is the amino acids for residues 471–585 of the protein. Identity of amino acids: *—identical, :—conservative, .—semi-conservative, space—non-conservative.

The *COI1* gene consisted of 603 amino acids in *S. lycopersicum* (NP_001234464.1) plants. Part of the *COI1* homologs sequenced in *Ribes* spp. consisted of 114 amino acids at the 3′ end of the *COI1* gene. Four heterogeneous isolates—COI_4.2.7, COI_5, COI_8 and COI_9—were submitted to the NCBI database with the accession numbers OK625547–OK625550, respectively (Table S5). Multiple alignments among sequences showed relatively high homology with part of the *COI1* gene in *S. lycopersicum* (Figure 5); 73.7% of identical and 11.4% of conservative amino acids were detected. Two conservative leucine-rich repeat (LRR) regions in the *R. nigrum* sequences were determined. Between sequences, semi-conservative (8.8%) and non-conservative (6.1%) amino acid substitutions were detected, and Glycine (G) deletion in *R. nigrum* isolates were found.

**Figure 6:**
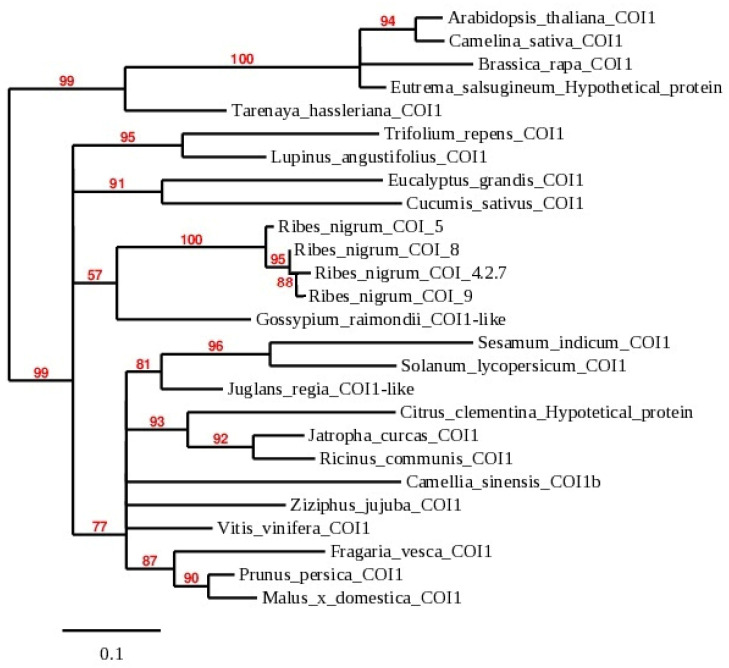
Phylogenetic dendrogram of *COI1* genes constructed from nucleotide sequences of 22 plant species. Accession numbers of newly obtained *COI1* sequences in *R. nigrum* in our research are OK625547—OK625550 (Table S5); accession numbers of other plants in Figure 6 are available in Table S2.

In the phylogenetic tree, 22 nucleotide sequences from various plants (Figure 6), including four identified isolates in the *R. nigrum* genome, showed dependence on the *COI1* family. All *COI1* homologs presented in the dendrogram were divided into two branches at 99.0% bootstrap, and they revealed the arrangement of *R. nigrum COI* homologs COI_5, COI_4.2.7, COI_8 and COI_9 in the separate branch in the second clade of the dendrogram. *R. nigrum* isolates were heterogeneous, and identity among them ranged from 95.9 to 98.8% (Table S4) in amino acid level.

**Figure 7:**
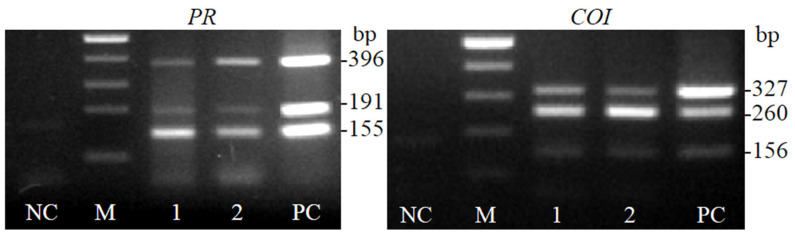
PCR products amplified with newly designed primer pairs specific to *R. nigrum PR*s, *PR1*, *PR6* and *COI1* genes. NC—negative control; M—size standard 100, 200, 300, 400, 500 bp; 1—cDNA of cv. Aldoniai; 2—cDNA of cv. Ben Tirran; PC—positive control.

According to the distribution of the *R. nigrum PR1, PR6* and *COI1* homologs in the phylogenetic trees (Figure 4 and Figure 6) and data of multiple sequence alignments (Figure 3 and Figure 5), six pairs of specific primer pairs were generated (Table 2). Primers were approved for DNA synthesis initiation by PCR in two *R. nigrum* cultivars: Aldoniai and Ben Tirran (Figure 7). The primer pair PRP_2847 allowed us to amplify a 155 bp-specific fragment of putative *PR6* in *R. nigrum* cDNA. According to the sequences of isolates (PRP_1, PRP_3 and PRP_9), the primer pair PRP_913 was generated, which flanked the fragment of 191 bp in length of the putative *PR1* sequence. The primer pair Ribes_PRP was suitable for *PR*s detection in *Ribes* spp. plants, and a specific fragment of 396 bp in length was amplified (Figure 7).

Although all newly detected *COI* homologs in *R. nigrum* belong to the *COI1* family, the nucleic and amino acid differences between them led to the creation of different primers. *R. nigrum* isolates COI_5 and COI_247 differ in their phylogeny; thus, an individual primer pair for each of them was designed. The specific fragment of 260 bp in length was obtained with the primer pair COI_5 (Table 2), while a fragment of 327 bp in length was obtained using the primer pair COI_247. Ribes_COI was a general primer pair for identification of *COI1* in blackcurrants (Figure 7).

## 3. Discussion

Abundant studies on *PR* and *COI1* homologs in many economically important crop cultures and modeling plants have been carried out [15,20,21]. The *Ribes* genome, belonging to the taxon *Saxifragales*, needs further study, and the *PR*s and *COI1* genes have also not been identified until now. The actions of these genes in different defense pathways in plants has been approved [14,15,16]. Based on the conservatism and ancient phylogenetic origin of these genes, their functions are likely to be similar in *Ribes* spp. to those of other plants. Phylogenetic analysis in this study provided evidence that *R. nigrum* contains *PR1*, *PR6* and *COI1* homologs with heterogeneity at nucleic acid and amino acid levels (Figure 3 and Figure 5).

*R. nigrum PR* homologs from families *PR1* (isolates PRP_1, PRP_3 and PRP_9) and *PR6* (isolates PRP_2, PRP_4, PRP_7 and PRP_8) were found, which confirms the presence of the CAP domain in protein sequences and the data of phylogenetic analysis (Figure 3 and Figure 4). Proteins of families of PR accumulate after the interaction of pathogens and may act as antifungal agents in other plants [22]. It is known that *PR*s’ activity is induced by biotic stress, and that they play an important role in plant defense against pathogenic fungi, viruses, bacteria and insects [23]. After reviewing aspects of this *PR* study, it was observed that the expression of *PR1* genes has been extensively studied in response to different types of insects and pathogen attacks in various plant species: fungus in rice [24], fungus, viruses and insects in wheat [25], bacteria in apple [26], etc. *PR1* is the most abundant family, and the expression of genes is usable as a molecular marker to indicate plant defense response [27]. The action of the *PR1* family members during viral infection is also known [21]. According to the literature, members of the *PR6* family are proteinase inhibitors related to defense response against insects, herbivores, microorganisms and nematodes [28]. It has been suggested that *PR6* can be important in plant response to abiotic stresses such as heavy metals, salt, water deficit or mechanical wounding [20,29,30,31]. It was determined that the biotrophic pathogen activates the SA-signaling pathway in plants that stimulates the accumulation of *PR1*, *PR2* and *PR5* products that systematically lead to SAR and can act locally [32]. In contrast, insufficient studies were conducted on *PR6*. The identification and characterization of the *PR6* family have been demonstrated in Gramineae [33], Leguminosae [34] and Solanaceae families [35], and expression analysis was performed in *P. ginseng* in response to signaling molecules to abiotic stresses [36].

All the *COI1* homologs identified in blackcurrants in this study belong to the *COI1* family; however, the *Ribes* genus has an individual phylogenetic way in species evolution (Figure 6). *COI1* is a key regulator involved in the wound or JA-signaling pathway required for plant-defense response [19]. Similar studies were performed, providing data that transcription of *COI1* differs in response to nematode infection in white clover [37], fungus infection in wheat [38] and virus infection in tobacco [39] and rice [40].

Newly designed primer pairs with degenerate oligonucleotides for *Ribes* genus and specific primers for *R. nigrum* were validated and are suitable for the identification of *PR*s, *PR1*, *PR6* and *COI1* homologs by PCR (Table 1 and Table 2, Figure 7). However, in this study, we have not evaluated the expression of these genes, so we can only assume the function and relevance of these genes for *Ribes* plants. To elucidate the role of these protective genes in blackcurrants, it is necessary to carry out further experiments on genes’ expression. For this purpose, stress factors must be modified and applied to plants in an in vitro system. Sequencing, comparative and in silico analysis revealed and evaluated the origin, diversity and mutations of partial *PR*s and *COI1* genes in *R. nigrum* plants. Now, we can develop a strategy for genetic engineering of the *R. nigrum* in vitro through overexpression of *PR* and *COI* proteins’ coding genes. The development and validation of specific primers suitable for the identification of the putative *PR1*, *PR6* and *COI1* genes of blackcurrants expand the possibilities for explaining the significance of these genes in *Ribes* spp. plants during biotic and abiotic stress.

## 4. Materials and Methods

### 4.1. Plant Material, RNA Extraction and cDNA Synthesis

Total RNA from homogenized leaves of *R. nigrum* cv. Didikai, *R. americanum*, *R. pauciflorum*, *R. hudsonianum*, *R. sanguineum*, *R. glandulosum*, *R. dikusha*, *R. uva-crispa* and two blackcurrant cultivars, Ben Tirran and Aldoniai, was extracted using GeneJET Plant RNA Purification Mini Kit (Thermo Scientific, Vilnius, Lithuania). Isolated RNA was used to synthesize cDNA using Maxima H Minus First Strand cDNA Synthesis Kit (Thermo Scientific, Vilnius, Lithuania). The concentration of cDNA was measured with an Implen GmbH spectrophotometer and stored at −20 °C until the reaction.

### 4.2. Primers Design and Polymerase Chain Reaction (PCR)

Degenerate oligonucleotide primer pairs for detection of *PR* and *COI* by PCR were designed using the Primer3plus program [41]. Primers were selected from the conservative sites of the genes by comparing sequences of other plants from the NCBI database. Alignments of the 18 *PR1* and 22 *COI1* nucleotide sequences were performed (Table S1, Figures S1 and S2).

Fragments of putative genes *PR1* and *COI1* in selected plant material were amplified using the PCR method. PCR was performed in a 20.0 µL reaction volume containing 11.1 μL H_2_O, 2.5 μL *Taq* + KCl buffer, 2.0 μL 2mM dNTP mix, 2.0 μL MgCl_2_, 2.0 µL cDNA, 0.2 μL *Taq* polymerase (Thermo Scientific, Vilnius, Lithuania) and 0.1 μL of each forward and reverse primer. The DNA was amplified using the following thermocycling steps: 95 °C for 3 min; 35 cycles of denaturing at 94 °C for 30 s; annealing (temperatures are given in Table 1 and Table 2) for 30 s; extending at 72 °C for 40 s; followed by a final step at 72 °C for 5 min and hold at 4 °C. The PCR products were resolved by electrophoresis through a 1.3% agarose gel and visualized by ethidium bromide staining and UV illumination.

### 4.3. Fragment Purification, Cloning and Sequencing

The amplified *R. nigrum* cv. Didikai cDNA fragments were excised from agarose gel and purified using the GeneJET Gel Extraction Kit (Thermo Scientific, Vilnius, Lithuania) according to the manufacturer’s protocol. Fragments were ligated into the pJET 1.2 blunt vector using the CloneJET™ PCR Cloning Kit (Thermo Scientific, Vilnius, Lithuania). Bacteria *Escherichia coli* JM107 were transformed with the TransformAid Bacterial Transformation Kit (Thermo Scientific, Vilnius, Lithuania). A total of 20 plasmids with cDNA inserts of *PR* and *COI* homologs were prepared for sequencing using the Big Dye Terminator v 3.1 Cycle Sequencing Kit and performed on a 3130 Genetic Analyzer Gene Analyzer (Applied Biosystem, Waltham, MA, USA). The sequenced 7 *PR* and 4 *COI* nucleotide sequences from *R. nigrum* were submitted to the NCBI database, and the accession numbers OK625407–OK625413 and OK625547–OK625550, respectively, were assigned (Table S5).

### 4.4. Statistical Analysis

The *PR1* and *COI1* sites, from which primers with degenerate nucleotides were generated, were visualized using the UGENE program (Kalign alignment) [42]. The multiple amino acid sequences’ alignments of *PR*s and *COI1* were performed with ClustalW (MUSCLE 3.8) [43] according to the *S. lycopersicum COI1* sequence (accession number NP_001234464.1 in the NCBI database) and the *V. vinifera PR1* sequence (accession number XP_002273416.1 in the NCBI database). The percent identity matrixes at amino acid and nucleic acid levels among the genes identified in *R. nigrum* were created using the Clustal 2.1 program. The phylogenetic dendrograms among the different plants (Table S2) and the obtained sequences in *R. nigrum* (Table S5) were constructed using the maximum likelihood method implemented in the PhyML program; a bootstrap analysis with 100 replications was performed [44].

## Figures and Tables

**Table 1:** Selected primer pairs with degenerate nucleotides for *PR* and *COI* detection.

Primer	Orientation	Oligonucleotide Sequences 5′ to 3′	Temperature, °C	Length, bp	Position in the Gene, bp
PRPd	Forward	GCMCARRAYWCHCCMCAAGAYT	63	392	66–458
	Reverse	TTGCCNSGDGGATCRTAAYTGCA			
COId	Forward	AGYCMAAAYGTRAGATGGATGCT	61	352	1383–1735
	Reverse	TTCTYKGWCCWGCHAGDGARTAR			

**Table 2:** Specific *PR* and *COI* primer pairs for *Ribes* spp.

Primer	Orientation	Oligonucleotide Sequences 5′ to 3′	Temperature, °C	Length, bp
PRP_2847	Forward	AGCACAAGTTGGTGTTGCAG	60	155
	Reverse	TAAAAGAACTACCGCTGCTCATT		
PRP_913	Forward	CTTGGGGAAGTGGTGAACTAAC	59	191
	Reverse	ATGGAGGAACATTTATCGGATG		
Ribes_PRP	Forward	CCCAGGACTCACCCCAAGATT	63	396
	Reverse	TGCCTGGGGGATCGTAATTG		
COI_5	Forward	AGCCTTCAGAAACTGGAATTGA	60	260
	Reverse	GCCAGGGAGTAATATGCTAGTATATGT		
COI_247	Forward	AGTCCAAACGTGAGATGGATGCTT	63	327
	Reverse	AGCCCAAACGTAAGATGGATGCT		
Ribes_COI	Forward	CACCTGACTGCTCTGAGGTACTTA	60	156
	Reverse	AACGACTACAGGTTCCTCTCCTT		

## Data Availability

The study did not report any data.

## Supplementary Materials

https://www.mdpi.com/article/10.3390/plants11030355/s1, Table S1: Plant species and their accession numbers from NCBI database, used in degenerate *COI* and *PR* primers; Figure S1: Multiple alignment of *PR1*; Table S2: Plant species and their accession numbers from NCBI database, used for *COI* and *PR* phylogenetic analysis; Figure S2: Multiple alignment of *COI1*; Table S3: Percent identity matrix of *PR* isolates at nucleic acid and amino acid levels identified in *R. nigrum* cv. Didikai; Table S4: Percent identity matrix of *COI* isolates at nucleic acid and amino acid levels identified in *R. nigrum* cv. Didikai; Table S5: The sequenced 7 *PR* and 4 *COI* isolates and their accession numbers identified in blackcurrant mRNA.
