# Supplementary material for: Putative Genes of Pathogenesis-Related Proteins and Coronatine-Insensitive Protein 1 in Ribes spp
Source: Plants (Basel). 2022 Jan 28;11(3):355. doi: 10.3390/plants11030355 (PMC8838371; doi:10.3390/plants11030355)
Supplement: Supplementary file 1 [file plants-11-00355-s001.zip › Supplementary files/Supplementary files.pdf]

Table S1. Plant species and their accession numbers from NCBI database, used in degenerate *COI* and *PR* primers design

Nr.	*PR*	*COI*
1	AF053343.2 *Capsicum annuum*	NM_129552.4 *Arabidopsis thaliana*
2	XM_016037449.1 *Ziziphus jujuba*	XM_010507391.2 *Camelina sativa*
3	XM_019000618.1 *Juglans regia*	XM_006411175.1 *Eutrema salsugineum*
4	XM_009380317.2 *Pyrus × bretschneideri*	XM_009135144.2 *Brassica rapa*
5	XM_008800672.2 *Phoenix dactylifera*	XM_010556341.2 *Tarenaya hassleriana*
6	XM_006486759.2 *Citrus sinensis*	XM_018980591.1 *Juglans regia*
7	XM_008348127.2 *Malus × domestica*	XM_007220373.2 *Prunus persica*
8	JQ765389.1 *Ficus pumila*	XM_016021798.1 *Ziziphus jujuba*
9	XM_007201223.2 *Prunus persica*	XM_008394693.2 *Malus × domestica*
10	XM_019855721.1 *Elaeis guineensis*	XM_012232540.1 *Jatropha curcas*
11	KC907719.1 *Actinidia eriantha*	XM_006435607.1 *Citrus clementina*
12	XM_010693219.2 *Beta vulgaris*	XM_012603983.1 *Gossypium raimondii*
13	XM_006379094.1 *Populus trichocarpa*	KU892070.1 *Camellia sinensis*
14	KM514665.1 *Hevea brasiliensis*	JQ281907.1 *Vitis vinifera*
15	XM_004289651.2 *Fragaria vesca*	XM_004150179.2 *Cucumis sativus*
16	XM_002273752.3 *Vitis vinifera*	XM_011078433.1 *Sesamum indicum*
17	KC551944.1 *Coffea canephora*	XM_019605965.1 *Lupinus angustifolius*
18	XM_008456842.2 *Cucumis melo*	XM_010069196.2 *Eucalyptus grandis*
19		KF022210.2 *Trifolium repens*
20		XM_002530373.2 *Ricinus communis*
21		NM_001247535.1 *Solanum lycopersicum*
22		XM_004307565.2 *Fragaria vesca*

Table S2. Plant species and their accession numbers from NCBI database, used for *COI* and *PR* phylogenetic analysis

Nr.	*PR*	*COI*
1	NM_127025.3 *Arabidopsis thaliana* (PR1)	NM_129552.4 *Arabidopsis thaliana* (COI1)
2	XM_043129043.1 *Carya illinoinensis* (PR1-like)	XM_010507391.2 *Camelina sativa* (COI1)
3	XM_018971312.2 *Juglans regia* (PR1A-like)	XM_006411175.1 *Eutrema salsugineum* (Hypothetical protein)
4	KR150952.1 *Hevea brasiliensis* (PR1)	XM_009135144.2 *Brassica rapa* (COI1)
5	XM_011031976.1 *Populus euphratica* (PR1-like)	XM_010556341.2 *Tarenaya hassleriana* (COI1)
6	XM_007041360.2 *Theobroma cacao* (PR1)	XM_018980591.1 *Juglans regia* (COI1-like)
7	XM_024307232.2 *Rosa chinensis* (PR1)	XM_007220373.2 *Prunus persica* (COI1)
8	XM_006422651.2 *Citrus clementina* (PR1)	XM_016021798.1 *Ziziphus jujuba* (COI1)
9	XM_009380317.2 *Pyrus* x *bretschneideri* (PR1-like)	XM_008394693.2 *Malus × domestica* (COI1)
10	XM_031088778.1 *Quercus lobata* (PR1-like)	XM_012232540.1 *Jatropha curcas* (COI1)
11	XM_002522019.3 *Ricinus communis* (PR1)	XM_006435607.1 *Citrus clementina* (Hypothetical protein)
12	XM_010271971.2 *Nelumbo nucifera* (PR6-like)	XM_012603983.1 *Gossypium raimondii* (COI1-like)
13	XM_010527344.1 *Tarenaya hassleriana* (PR6-like)	KU892070.1 *Camellia sinensis* (COI1b)
14	XM_006393121.1 *Eutrema salsugineum* (PR6)	JQ281907.1 *Vitis vinifera* (COI1)
15	XM_002274271.4 *Vitis vinifera* (PR6)	XM_004150179.2 *Cucumis sativus* (COI1)
16	XM_007201550.2 *Prunus persica* (PR6)	XM_011078433.1 *Sesamum indicum* (COI1)
17	XM_008238015.1 *Prunus mume* (PR6-like)	XM_019605965.1 *Lupinus angustifolius* (COI1)
18	XM_028246868.1 *Camellia sinensis* (PR6-like)	XM_010069196.2 *Eucalyptus grandis* (COI1)
19	XM_022133207.2 *Helianthus annuus* (PR6)	KF022210.2 *Trifolium repens* (COI1)
20	XM_016587507.1 *Nicotiana tabacum* (PR6-like)	XM_002530373.2 *Ricinus communis* (COI1)
21	XM_002522025.2 *Ricinus communis* (PR6)	NM_001247535.1 *Solanum lycopersicum* (COI1)
22	XM_035071874.1 *Populus alba* (PR6-like)	XM_004307565.2 *Fragaria vesca* (COI1)

Table S3. Percent identity matrix of *PR* isolates at nucleic acid and amino acid levels identified in *R. nigrum* cv. Didikai

		Nucleic acid						
		PRP_4	PRP_2	PRP_7	PRP_8	PRP_1	PRP_3	PRP_9
Amino acid	PRP_4	*****	98.07	98.34	97.79	66.57	66.86	66.01
	PRP_2	98.33	*****	98.18	97.92	67.47	67.47	66.40
	PRP_7	98.33	98.43	*****	98.24	67.10	68.12	67.61
	PRP_8	97.50	97.64	98.48	*****	67.61	67.87	67.35
	PRP_1	64.96	63.71	65.12	65.89	*****	96.92	95.37
	PRP_3	65.81	66.13	65.89	65.12	95.35	*****	94.60
	PRP_9	63.25	63.71	65.12	65.89	92.25	92.25	*****

Table S4. Percent identity matrix of *COI* isolates at nucleic acid and amino acid levels identified in *R. nigrum* cv. Didikai

		Nucleic acid			
		COI_4.2.7	COI_5	COI_8	COI_9
Amino acid	COI_4.2.7	*****	95.86	97.94	97.16
	COI_5	91.96	*****	97.34	96.15
	COI_8	95.54	96.43	*****	98.82
	COI_9	94.87	94.64	98.21	*****

Table S5. The sequenced 7 *PR* and 4 *COI* isolates and their accession numbers identified in blackcurrant mRNA (submitted to the NCBI on October 25, 2021; our records will be released to the public database once they are processed)

Isolate	Accession nr.	Nucleotide sequence
PRP_1	OK625407	GCACAAGACTCACCCCAAGATTTCCTCGATGCCCACAACGCGGCTCGCTCTGCGGTTGGCGTAGG<br>GCCTATGTCATGGGACGCCACGGTAGAGTCTTACGCACAAAACTACGCTAATTCACGTAAAGTCG<br>ATTGCTATATGGTGCACTCCAACGGTCCTTACGGCGAGAACATTGCTTGGGGAAGTGGTGACCTA<br>ACGGAAACAAGTGCTGTCAAAATGTTCGTAGATGAGAAGGTTAACTATAACCATGACTCGAATTC<br>GTGTGTAGGAGGAGAGTGTCGACATTATACACAAGTTGTTTGGCGAGACTCCGTTCATCTTGGGT<br>GTGCTGAAGTGCGATGCAACAATGGAGGGACATTTATCGGATGCAATTACGATCCTCGGGGCAA
PRP_2	OK625408	CCAAGATTACCTCAATTCTCACAATGCAGCTCGAGCACAAGTTGGTGTTGCAGACATGATATGGG<br>ACGACACGGTGGCGGCTTACGCACAGAGCTATGCAAATCAAAGGATGGCCGATTGCGCGCTCATA<br>CACTCAAACTCGGCACTTTATGGCGAGAACATCGCAATGAGCAGCGGTAGTTCTTTTACGGGCAC<br>AGACGCCGTGAAGTTGTGGGTAGATGAGAAGCCTTACTATGACTACACCTCCAACTCTTGTACGG<br>GAGGAGAAGAGTGCTTGCATTATACTCAAGTTGTTTGGAGTGACTCGATTCGACTTGGATGTGCT<br>GGGGTTGAATGTAACAATGGTGGGTCTTTTGTTACTTGCAGTTATGATCCTCCTGGCAA
PRP_3	OK625409	GCCCAGAACACTCCCCAAGATTTCCTCGATGCCCACAACGCGGCTCGCTCTGCGGTTGGCGTAGG<br>GCCTATGTCATGGGACGCCACGGTAGCGTCTTACGCACAAAACTACGCTAATTCACGTAAAGTCG<br>ATTGCTATATGGTGCACTCCAACGGTCCTTACGGCGAGAACATTGCTTGGGGAAGTGGTGACCTA<br>ACGGAAACAAGTGCTGTCAAAATGTTCGTAGATGAGAAGGTTAACTATAACCATGACTCGAATTC<br>GTGTGTAGGAGGGGAGTGTCGACATTATACACAAGTTGTTTGGCGAGACTCCGTTCATCTTGGGT<br>GTGCTAAAGTGCGATGCAACAATGGAGGGACATTTATCGGATGCAGTTACGATCCCCCAGGCAA
PRP_4	OK625410	AATGCAGCTCGAGCACAAGTTGGTGTTGCAGACATGATATGGGACGACACGGTGGCAGCTTACGC<br>ACAGAGCTATGCAAATCAAAGAATGGCCGATTGCGCGCTCATACACTCAAACTCGGCACTTTATG

		GCGAGAACATCGCAATGAGCAGCGGTAGTTCTTTTACGGGCACAGACGCCGTGAAGTTGTGGGTA<br>GATGAGAAGCCTTACTATGACTACACCTCCAACTCTTGTACGGGAGGAGAAGAGTGCTTGCATTA<br>TACTCAAGTTGTTTGGAGTGACTCGATTCGACTTGGATGTGCTAGGGTTGAATGTAACAATGGTG<br>GGTCTTTTGTTACTTGCAGTTACGATCCCCGGGGCAA
PRP_7	OK625411	GCCCAGGACACTCCACAAGACTACCTCAATTCTCACAATGCAGCTCGAGCACAAGTTGGTGTTGC<br>AGACATGATATGGGACGACACGGTGGCGGCTTACGCACAGAGCTATGCAAATCAAAGGATGGCCG<br>ATTGCGCGCTCATACACTCAAACTCGGCACTTTATGGCGAGAACATCGCAATGAGCAGCGGTAGT<br>TCTTTTACGGGCACAGACGCCGTGAAGTTGTGGGTAGATGAGAAGCCTTACTATGACTACACCTC<br>CAACTCTTGTACGGGAGGAGAAGAGTGCTTGCATTATACTCAAGTTGTTTGGAGTGACTCGATTC<br>GACTTGGATGTGCTAGGGTTGAATGTAACAATGGTGGGTCTTTTGTTACTTGCAATTACGATCCA<br>CCCGGCAA
PRP_8	OK625412	GCCCAGGATTCACCACAAGATTACCTCAATTCTCACAATGCAGCTCGAGCACAAGTTGGTGTTGC<br>AGACATGATATGGGACGACACGGTGGCGGTTTACGCACAGAGCTATGCAAATCAAAGGATGGCCG<br>ATTGCGCGCTCATACACTCAAACTCGGCACTTTATGGCGAGAACATCGCAATGAGCAGCGGTAGT<br>TCTTTTACGGGCACAGACGCCGTGAAGTTGTGGGTAGATGAGAAGCCTTACTATGACTACACCTC<br>CAACTCTTGTACGGGAGGAGAAGAGTGCTTGCATTATACTCAAGTTGTTTGGAGTGACTCGATTC<br>GACTTGGATGTGCTAGAGTTGAATGTAACAATGGTGGGTCTTTTGTTACTTGCAATTACGATCCA<br>CCAGGCAA
PRP_9	OK625413	GCACAAGACTCACCCCAAGACTTCCTCGATGCCCACAATGCGGCTCGCTCTACGGTTGGCGTAGG<br>GCCTATGTCATGGGATGCCACGGTAGCGTCTTACGCACAAAACTATGCTAATTCACGTAAAGCCG<br>ATTGCTATATGGTGCACTCTAACGGTCCTTACAGCGAGAACATTGCTTGGGGAAGTGGTGAACTA<br>ACGGAAACAAGTGTTGTCAAAACGTTCGTAGATGAGAAGGTTAACTATAACCATGACTTGAATTC<br>GTGTGTAGGAGGAGAGTGTCGACATTATACACAAGTTGTTTGGCGAGACTCCGTTCATCTTGGGT<br>GTGCTAAAGTGCGATGCAACAATGGAGGAACATTTATCGGATGCAATTACGATCCCCCCGGCAA
COI_4.2.7	OK625547	AGTCCAAACGTGAGATGGATGCTTTTGGGCTATGTTGGTGAAACAGATCAAGGGCTTCTGGGATT<br>CTCAAGAGGCTGCCCCAGCCTTCAGAAACTGGAATTGAGGGGCTGTTGCTTCAGTGGGCATGCAC<br>TGGCCGTGGCTGTAATGCACCTGACTGCTCTGAGGTACTTATGGGTGCAAGGATACAGAGCATCT<br>GAAACAGGCCTTGATCTCCTAGCTATGGCTCGGCCTTTTTGGAATATTGAGCTCATTCCTGTAAG<br>ACGAATCATTGCTGCTGATCAAGGAGAGGAACCTGTAGTCGTTGAGCATCCATCTTACGTTTGGG<br>CT
COI_5	OK625548	ATGGATGCTTTTGGGCTATGTTAGTGAAACAGATCAAGGGCTTCTGGAATTCTCAAGAGGCTGCC<br>CCAGCCTTCAGAAACTGGAATTGAGGGGCTGTTGCTTCAGTGAGCATGCACTGGCCGTGGCTGCA<br>ATGCACCTGACTGCTCTGAGGTACTTATGGGTGCAAGGATACAGAGCATCTGAAACAGGCCTTGA<br>TCTCCTAGCTATGGCTCGGCCTTTTTGGAATATTGAGCTCATTCCTGCAAGACGAATCATTGCTG<br>CTGATCAAGGAGAGGAACCTGTAGTCGTTGAGCATCCAGCACATATACTAGCATATTACTCCCTG<br>GCTGGACAAAGA
COI_8	OK625549	GATGGATGCTTTTGGGCTATGTTGGTGAAACGGATCAAGGGCTTCTGGAATTCTCAAGAGGCTGC<br>CCCAGCCTTCAGAAACTGGAATTGAGGGGCTGTTGCTTCAGTGAGCATGCACTGGCCGTGGCTGT<br>AATGCACCTGACTGCTCTGAGGTACTTATGGGTGCAAGGATACAGAGCATCTGAAACAGGCCTTG<br>ATCTCCTAGCTATGGCTCGGCCTTTTTGGAATATTGAGCTCATTCCTGCAAGACGAATCATTGCT<br>GCTGATCAAGGAGAGGAACCTGTAGTCGTTGAGCATCCATCACACATTTGGGCT
COI_9	OK625550	AGCCAAAATGTGAGATGGATGCTTTTGGGCTATGTTGGTGAAACGGATCAAGGGCTTCTGGAATT<br>CTCAAGAGGCTGCCCCAGCCTTCAGAAACTGGAATTGAGGGGCTGTTGCTTCAGTGAGCATGCAC<br>TGGTCGTGGCTGTAATGCACCTGACTGCTCTGAGGTACTTATGGGTGCAGGGATACAGAGCATCT<br>GAAACAGGCCTTGATCTCCTAGCTATGGCTCGGCCTTTTTGGAATATTGAGCTCATTCCTGCAAG<br>ACGAATCATTGCTGCTGATCAAGGAGAGGAACCTGTAGTCGTTGAGCATCCATCTCACGTTTGGG
