# Supplementary figures and images for: Putative Genes of Pathogenesis-Related Proteins and Coronatine-Insensitive Protein 1 in Ribes spp
Source: Plants (Basel). 2022 Jan 28;11(3):355. doi: 10.3390/plants11030355 (PMC8838371; doi:10.3390/plants11030355)

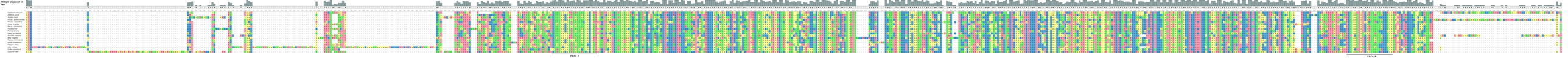

Supplement: Supplementary file 1 [file plants-11-00355-s001.zip › Supplementary files/Figure S1.jpg]
